# Influence of hydrogenated diesel/H_2_O_2_ blend fuel on diesel engine performance and exhaust emission characterization

**DOI:** 10.1038/s41598-023-27569-5

**Published:** 2023-01-16

**Authors:** Iqbal Ahmed Moujdin, Muhammad Saad Khan, Hani Abdulelah Abulkhair, Amer Ahmed Shaiban, Hussam Adnan Organji, Abdulmohsen Omar Alsaiari

**Affiliations:** 1 Center of Excellence in Desalination Technology, King Abdulaziz University, PO Box 80200, Jeddah, 21589 Saudi Arabia; 2 Department of Mechanical Engineering, King Abdulaziz University, PO Box 80200, Jeddah, Saudi Arabia; 3 CO2 Research Center, University Technology PETRONAS, Seri Iskandar, Malaysia

**Keywords:** Environmental sciences, Chemistry, Energy science and technology, Engineering, Materials science

## Abstract

The oxygenated hydro diesel (OHD) is prepared from hydrogen peroxide (H_2_O_2_), acetone, and seaweed polysaccharide. A long-term study was carried out on the OHD fuel blend stability for about a year at various temperatures. The long-term stability shows very stable properties, no easy emulsion breaking, and a long storage period. The neat diesel and blend fuel performance test was conducted at various engine speeds, 1700–3100 RPM the diesel blend with 5 wt.% and 10 wt. % of H_2_O_2_ revealed the best fraction for reducing smoke and emissions. The blend contains 15 wt.% H_2_O_2,_ revealing a significant reduction in exhaust temperature without considering the engine's performance. Moreover, the performance of the OHD also revealed an economizing rate, decreasing environmental pollution and prolonging the engine’s service life. The diesel engine performance and environmental evaluation leading to exhaust emissions characterization (${\mathrm{CO}}_{\mathrm{X}}$, ${\mathrm{SO}}_{\mathrm{X}}, {\mathrm{NO}}_{\mathrm{X}}$, and others). Based on the results, the various concentrations of H_2_O_2_ are an effective method for reducing the emission of diesel engines. Decreased CO, SO_2_, unburned hydrocarbons, and NO_2_ were also observed as percentages of H_2_O_2_. Due to increased oxygen content, water content and cetane number, the number of unburned hydrocarbons from diesel fuel decreased with the addition of H_2_O_2_. Therefore, the OHD blend can significantly curtail the exhaust emission of conventional diesel fuel, which will help reduce the harmful greenhouse gas emissions from diesel fuel sources.

## Introduction

In fossil fuel, diesel usage is quite significant, such as in transportation, heavy and light vehicles, shipping, and numerous agricultural and industrial practices^1,2^. Moreover, because of its remarkable energy potential, diesel fuels are also used in large-scale power generation and residential heating systems. The diesel engine is generally considered the most powerful of all the internal combustion engine types. Although the default calorific values of diesel fuel might be lower than other petroleum fuels, it has proven to have higher calorific proficiency in engine structure. In addition, diesel fuel leads an extraordinary command power, efficient fuel economy, and considerably higher lifecycle and consistency^3–5^.

Regardless of the best performance fuel, diesel is one of the most significant contributors of pollutants released by on-road and off-road vehicles and large-scale marine diesel engines^6,7^. As a result, much emphasis has been placed on improving diesel fuels, as well as theoretical and practical investigations into the relationship between NOx, COx, and hydrocarbon emissions, as well as condensed material emissions such as particulate matter (PM) and soot^8^. Emissions sourced from an engine are determined by operating conditions and the type of fuel utilized, as shown in Appendix 1, which provide the core emissions from diesel engines comprising NOx, SO_x_, CO, VOC, NO_2_, NO and CO_2_^6,9^.

**Figure 1:**
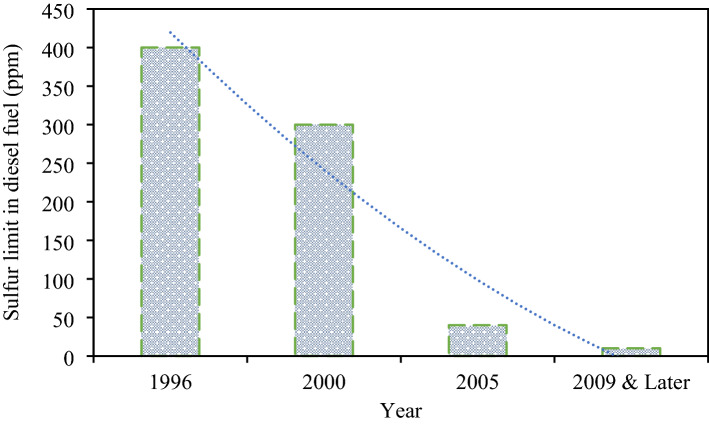
Recent limitations of sulfur contents in clean diesel fuel^11,12^.

Nevertheless, besides further emission factors, another critical factor is the sulfur contents in diesel fuel. Increased restrictions on diesel fuel have had a considerable consequence on scouring up the exhaust. The sulfur content of diesel fuel is now confined to 15 parts per million (ppm) when formerly it was as high as 400–550 ppm (EURO diesel I and EURO II)^10,11^. Figure 1 illustrates the current limitations of sulfur contents in clean diesel fuel.

The critical property of diesel fuel is its cetane number which affects ignition delay to combustion^13–15^. Fuel containing a higher cetane number enhances the combustion process during operation^16^. Yet, increasing concern over environmental protection and rigorous governmental regulations on exhaust emissions to reduce pollution has sparked a significant increase in engine development research^17^. Reducing particulate matter (PM) and ${\mathrm{NO}}_{\mathrm{X}}$ particularly in the Euro VI standards simultaneously is problematic owing to a converse relationship between ${\mathrm{NO}}_{\mathrm{X}}$ and PM^18^. Numerous researchers are dedicated to developing new or improved after-treatment technology to reduce ${\mathrm{NO}}_{\mathrm{X}},$ PM and non-methane volatile organic compounds (NMVOC) emissions^19–22^. Selective Catalytic Reduction (SCR) is the most sophisticated active emissions control technology utilized effectively in diesel engine vehicles^23,24^. SCR uses a monolith catalyst to convert NOx into water (H_2_O) and diatomic nitrogen (N_2_)^7^.

Due to sustainable development and environmental concern, substantial attention has been given to developing reformulated or alternative fuels. Many of these efforts have been focused on improving diesel fuel in the form of blended fuel to obtain durable and efficient superior blends to replace conventional diesel fuel. The primary diesel fuel blends developed thus far include ethanol^25–29^, biodiesel^30–33^, hydrogen^11,34,35^; water-diesel^2,36,37^, vegetable oil^38–40^ and various other oxygenated fuels^41–44^. It is widely thought that the reformulation of diesel fuels has played a significant role in attaining considerable reductions in exhaust emissions^39,45–47^. The reformulation of diesel fuels brought additional advantages, including lowering sulfur and aromatic contents and the possibility of adding oxygen to the fuel. Many oxygenates-based additives have proven quite effective in reducing particulate emissions from diesel engines^48–50^. However, the most significant problem with diesel fuel is its reduced ability to dissolve other fuel blends. Once an additive is inserted as an adjunct, a sudden reduction in fuel properties is observed, especially in the number of cetanes drops significantly^51^. Diesel fuel partially mixes with ethanol, but solubility is affected due to the difference in surface tensions for both liquids.

Water is a typical diesel fuel additive that can be combined with diesel to co-existence an emulsifier^52^. Furthermore, water can be sprayed directly into the combustion chamber or fumigated into the intake air^53^. Recently, Atarod et al.^54^ performed an experimental and modelling study on the nanoparticle-induced water-diesel emulsified fuel for emission control from the diesel engine. A mixture of Span 80 and Tween 80 was used for 5 wt.% while water content and nanoparticle composition varied between 0–3 wt.% and 0–150 μM, respectively. Findings revealed that adding water to a diesel fuel mitigated the unburned hydrocarbon emission and nanoparticle drops in the nitrogen oxide formation at moderate load conditions. Furthermore, the developed neuro-fuzzy-logic-based model effectively predicted the operating parameters and exhaust emissions from water-diesel blend fuel.

One of the best possible ways to introduce the oxygenate fuel is the insertion of H_2_O_2_ in the diesel fuel blend, which has a higher cetane number tendency with additional water molecule^42–44^. However, previous studies illustrated that phase separation occurs with time by adding H_2_O_2_ to an ethanol and diesel fuel solution^51^. Increased stability of the blend over a more extended period is also a significant issue^55^. Moreover, Few studies have indicated several potential uses for H_2_O_2_ in combustion processes with a broad range of energy conversion systems^13,44^. David & Reader^56^ and Golovitchev et al.^57^ studied the prospects of methane auto-ignition in the air with H_2_O_2_. They discovered that the ignition delay was significantly reduced by adding a small quantity of H_2_O_2_ (10% by volume). The ignition delay was reduced by order of magnitude for (i) CH_4_/O_2_/Air mixture at 2.55–13.01 atm, where the combustion temperature ranged from 1525 to 2025 K; and for (ii) CH_4_/air mixture at 0.4–10 atm with a temperature range of 1100–2000 K^56^. A subsequent study conducted by Golovitchev & Piliaf^57^ also found enhanced methane auto-ignition with H_2_O_2_ that was more resilient than lean hydrogen gas. This decreased ignition delay is understandably evident due to the role of ‘O’ and ‘OH’ radicals produced by the immediate decomposition of H_2_O_2_^57,58^.

Furthermore, Martinez et al.^59^ found that H_2_O_2_ catalyzed the conversion of lethal nitric oxide to less dangerous nitrogen dioxide in diesel exhaust. David & Reader^56^ and Ashok & Saravanan^51^ explained that a suitable injection of H_2_O_2_ into a diesel engine appreciably reduced soot and NO*x*. In addition, Martinez & Cabezas^59^ determined that concentrations of unburned hydrocarbons (NO*x* and CO) from an industrial pilot plant scale combustion chamber fueled with natural gas were significantly lowered by the injection of a few hundred ppm of H_2_O_2_. A supplementary study conducted by David & Reader^56^ showed that adding H_2_O_2_ reduced the CO concentration and NOx emissions, while Ashok & Saravanan^51^ demonstrated a rise in thermal brake efficiency. And Yusof et al.^44^ reported that increased H_2_O_2_ enhances the cetane number of diesel fuel blends significantly. Moreover, these studies also revealed lower specific fuel consumption, particulate matter, smoke density, nitrogen oxides of nitrogen, carbon monoxide, and hydrocarbons compared to diesel fuel on its own or mixed with emulsified fuel^51^.

Therefore, the present work focuses on studying the performance and emission characteristics of 5−15 wt.% added to diesel in the presence of a newly prepared polysaccharide polymer (agarose)/acetone emulsifier. In addition, the results are compared to the reference diesel (neat diesel). Our earlier study found the coherent stability of emulsified fuel. The experimental study also revealed that the increased H_2_O_2_ contents in the diesel significantly enhanced the cetane number of fuel blends. Hence, the present work is a continuation of our previous study to investigate the influence of hydrogenated diesel/${\mathrm{H}}_{2}{\mathrm{O}}_{2}$ blend fuel on diesel engine performance and exhaust emission characterization, particularly in reducing NO*x*, CO, ${\mathrm{C}}_{\mathrm{x}}{\mathrm{H}}_{\mathrm{y}}$, and ${\mathrm{SO}}_{2}$.

## Results and discussion

### Comparison of comprehensive output energy at various speed

**Figure 2:**
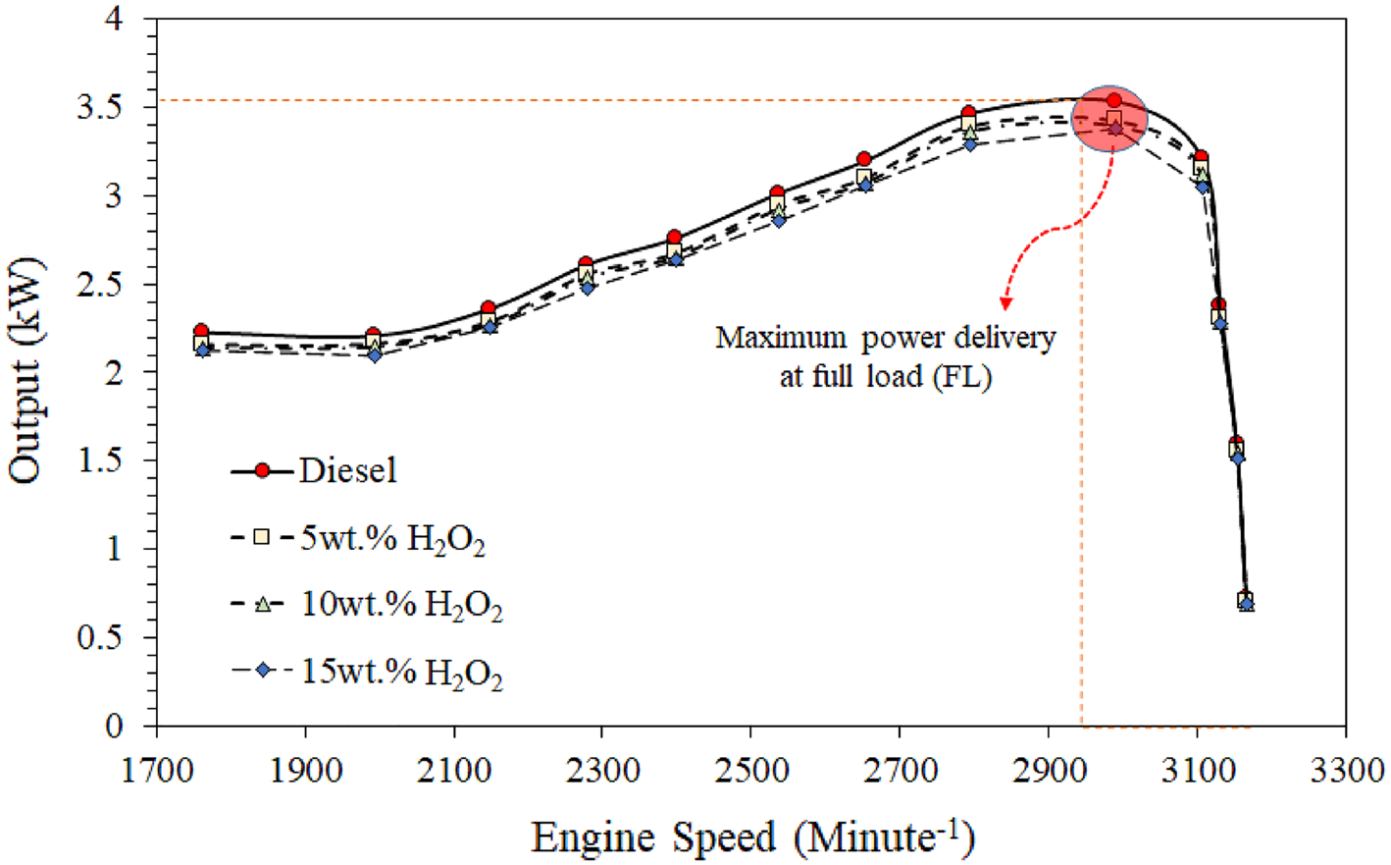
Comparison of power output efficiency of various quantities of H_2_O_2_/diesel fuel blends.

The output energy (OPE) at various speeds (rpm) is a tool for comparing the comprehensive performance of ${\mathrm{H}}_{2}{\mathrm{O}}_{2}$/diesel blend fuel with reference diesel (RD). Theoretically, it has measured how much fuel is being disbursed per breakup time to deliver maximum power. Figure 2 illustrates the generator output (kW) of the various test fuels at different engine speeds and various engine torque (6−12.5 Nm).

The results revealed that the RD fuel produced higher output at various engine speeds, almost 1–2.5%. However, emulsified H_2_O_2_/diesel blends showed lower output efficiency. The reason may be due to the relatively lower calorific value of H_2_O_2_/diesel fuel than the RD fuel discussed in our previous study^44^.

Amongst the ${\mathrm{H}}_{2}{\mathrm{O}}_{2}$/diesel blend fuel, the 5 wt.% of ${\mathrm{H}}_{2}{\mathrm{O}}_{2}$ showed somewhat higher than higher H_2_O_2_/diesel blend content. Such negligible deficiency could be revealed due to their higher combustion efficiency and effective oxygen content in the diesel blend fuel, which is perhaps a good agreement for early combustion efficiency compared to RD diesel. Moreover, in our previous work^13,44,60^, we have already demonstrated that adding H_2_O_2_ in the diesel enhanced the cetane number with thermal conductivity and specific heat. Perhaps the calorific value of the H_2_O_2_/diesel blend fuel scarcely lowered because of the lower energy contents of the fuel blends, despite all significances being agreeably within the scope of diesel fuel^47,61^.

### Specific fuel consumption (g/kWh)

**Figure 3:**
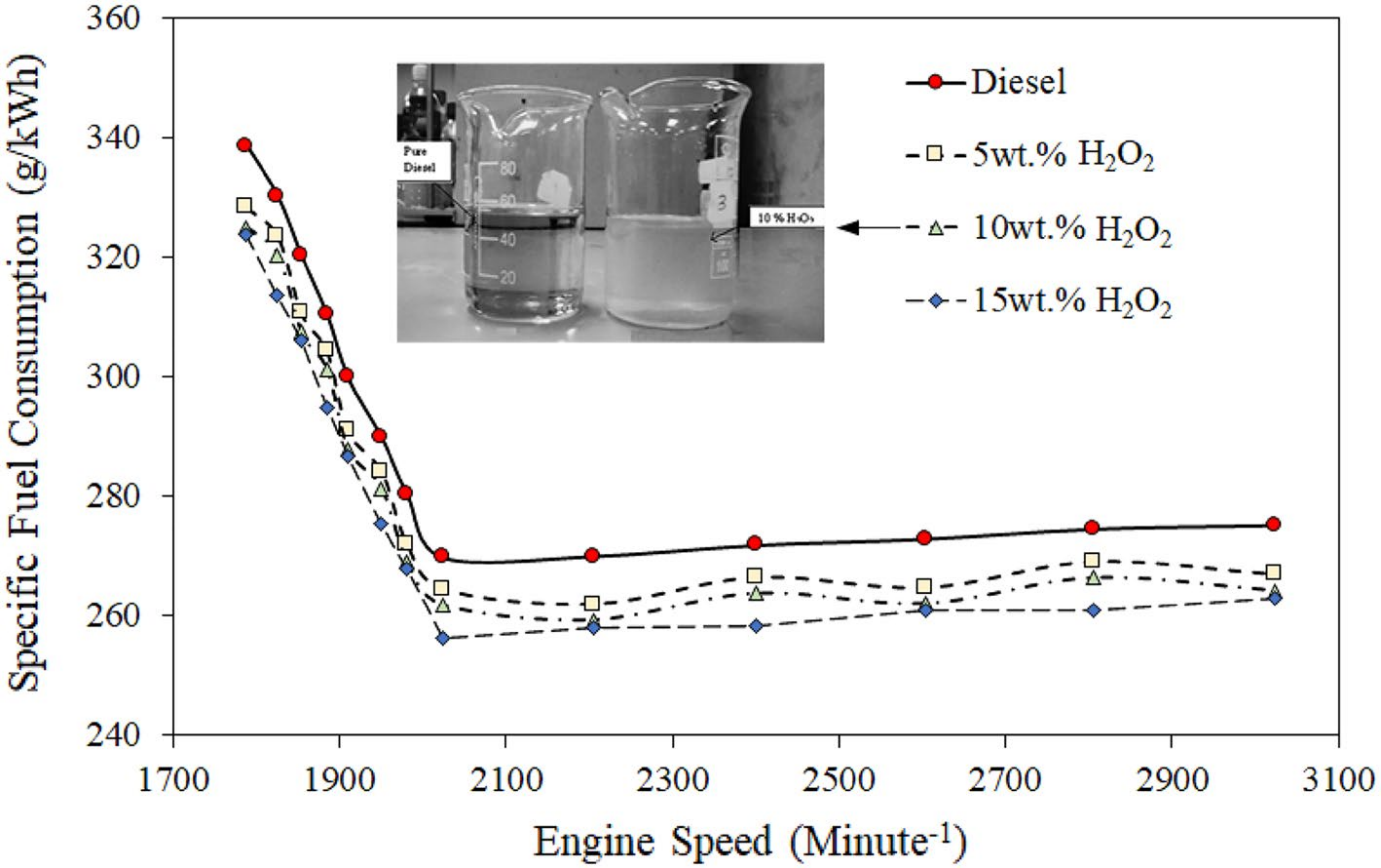
Comparison of specific fuel consumption efficiency of various quantities of H_2_O_2_/diesel fuel blends.

The current section of the study investigates engine performance using a convenient parameter of specific fuel consumption (SFC) and a comparison of the RD and H_2_O_2_/diesel blend fuel. The tests were conducted under various engine torque (6 12.5 Nm) and speed conditions ranging from 1700 to 3024 rpm. SFC indicates the ratio of fuel consumption rate to brake power output. Figure 3 summarizes the SFC of RD and diesel blend fuels; the results illustrated a decreasing trend as the engine speed increased from 1700 to 3024 rpm.

Because the test engine’s fuel injection pump was of a customized type, the delivered fuel quantity decreased at the minimum default speed of Yanmar^62^, such as 1700 rpm. They simulated the breakup comparison rate regarding the RD fuel–air mixing rate and excess oxygen content within the diesel blend fuel. Increasing the engine speed improved system performance while decreasing the SFC of each test fuel.

Nonetheless, the reduced volumetric coherence at higher speeds can reveal an SFC deficiency at speeds above 1700 rpm^62^. On average, the SFC of the RD test was higher than that of all H_2_O_2_/diesel blend fuels. RD fuel’s SFC was 2–5% higher than H_2_O_2_/diesel. The test fuel contains 10 and 15 wt.% H_2_O_2_, respectively, and the diesel blend showed more promising SFC results than the diesel blend with 5 wt.% H_2_O_2_. H_2_O_2_ demonstrated a 1.5 to nearly 5.2% reduction in SFC when compared to 5 wt.% H_2_O_2_/diesel and RD fuel, respectively. The higher SFC of the RD fuel than all H_2_O_2_/diesel blend fuels is attributed to the RD diesel’s slightly higher energy scope. Technically, the heating values of the fuel blends were lower due to the molar volume contents of H_2_O_2_ and emulsifier (C_14_H_24_O_9_/C_3_H_6_O); thus, consumption was supposed to be increased to achieve slightly more than 11 Nm torque. Despite having relatively lower heating values, all H_2_O_2_/diesel blend fuels had lower SFC than RD fuel. The reason for effective SFC is due to the higher cetane value of the H_2_O_2_/diesel fuel blend^51^. When the cetane number of blend fuel rises with increased quantities of H_2_O_2_, the temperature and oxygen content in the combustion chamber are in more self-control, promoting thermal cracking and increasing oxidation rates while decreasing unburned HC emissions and specific fuel consumption^63^. It also suggests that adequate SFC of the H_2_O_2_/diesel blends is perhaps found because of the presence of stable high oxygen contents in the diesel blend.

### Smoke density (SD)

The exhaust smoke density, also called multiple particulate matter (PM), relates to unburnt hydrocarbons (H_x_Y_x_), NO_x_, and SO_x_ and has proven to be a critical issue for diesel fuel. Therefore, since the last decay, developed countries have made rigorous policies to restrict light-grade diesel (EURO II and III) usage in public automobiles. Yet the PM, particularly H_x_Y_x_ and NO_x_, are still challenging in European countries due to the freezing environment^10,12^. Even though public transport uses high-speed diesel (EURO V and VI) followed by advanced technology like in-cylinder and advanced hybrid oxidation catalysts with catalytic filters system.

**Figure 4:**
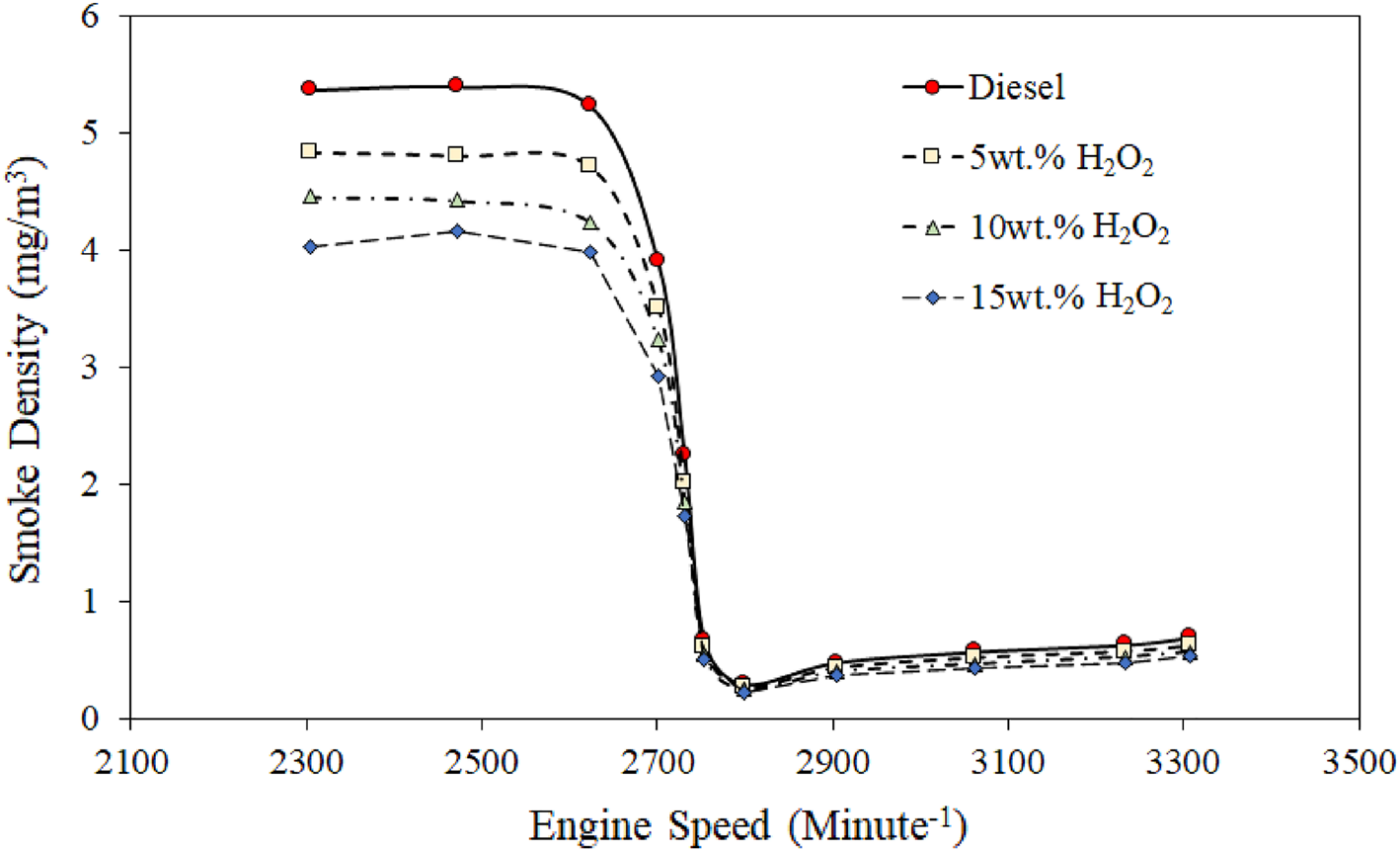
Comparison of exhaust smoke density of various quantities of H_2_O_2_/diesel fuel blends.

Thus, this section investigated a comprehensive assessment of the engine performance on the SD of different H_2_O_2_/diesel blend fuels. The SD analysis was carried out using an AVL smoke meter during the test running condition with variable torque (6−12.5 nm) followed by different engine speeds ranging from ~ 1700 to 3600 rpm. The SD results can be seen in Fig. 4; the SD comparison of H_2_O_2_/diesel blends with RD fuel showed a decreasing tendency as the engine speed increased from 1700 to 3600 rpm.

It has also been noticed that the SD followed a similar trend level to Fig. 4 decreases for each H_2_O_2_/diesel blend than the RD. However, the drought of SD showed a significant drop in all H_2_O_2_/diesel blend fuel, about 10–25% reductions. The reduction of SD level was probably revealed due to excess oxygen content, which has also been attributed to better mixing of intake air and fuel and an increase in the OH radical molar mass contents in the combustion chamber^38,51,64^. Usually, the components of diesel fuel exhibit an intense interaction capability with oxygen. Furthermore, the stability of diesel/H_2_O_2_ is higher, secondary combustion is reduced, and combustion performance is enhanced.

**Figure 5:**
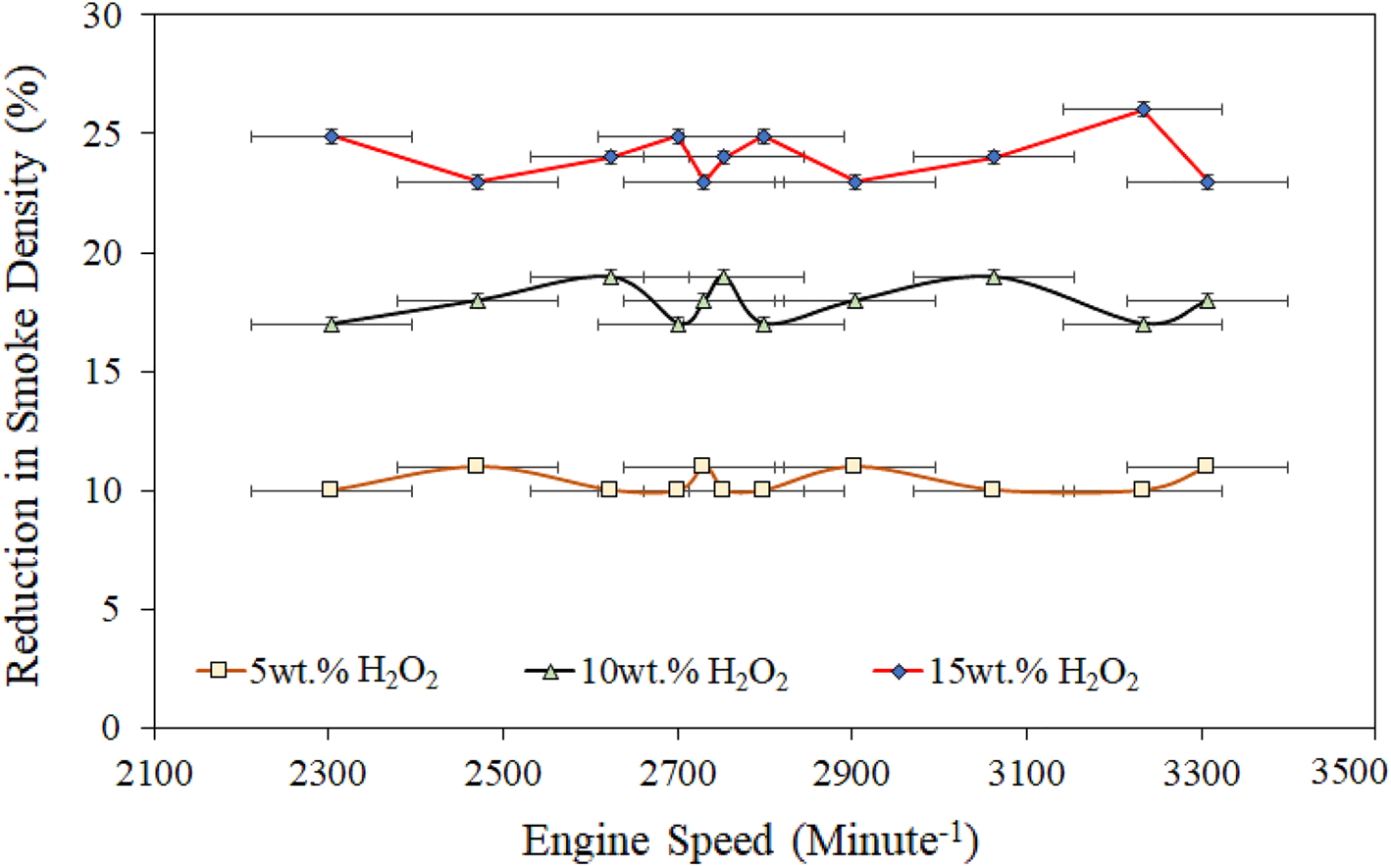
Comparison of percentage reduction in exhaust smoke density (SD) for various quantities of H_2_O_2_/diesel fuel blends.

Moreover, our previous studies have demonstrated that the emulsifier used in H_2_O_2_ and diesel prevented the phase rift between diesel and H_2_O_2_, as seen in Fig. 4^44^. Therefore, H_2_O_2_ likely invariably reduces soot and PM emissions in diesel. Also, it could be the consequence of rapid fuel breaking up due to the distinct engagement of oxygen content in the fuel combustion chamber, probably more related to smoke density. The highest SD reduction was obtained by 15 wt. of H_2_O_2_ diesel blend fuel at maximum load conditions is 26% (see Fig. 5).

Also, Fig. 5 shows the lower peak value attained by the 5 wt.% of H_2_O_2_ in the diesel blend fuel at a load speed of 2900 rpm is about 12%. The smoke density is further decreased with the 10 wt.% H_2_O_2_ addition in diesel blend because of excess oxygen content. Thus, it presumably revealed the molar volume difference between the agar/acetone (C_14_H_24_O_9_/C_3_H_6_O) and the ${\mathrm{H}}_{2}{\mathrm{O}}_{2}$ added diesel blend fuel, which might reveal the direct relevancy of SD and particulate matters (H_x_Y_x_ + No_x_) to each other. Particulate reduction will most likely be due to a good agreement in the combination of acetone and H2O2 in diesel fuel, which may act as an oxidizing agent to keep the combustion chamber clean. In addition, the SD is reduced for the H_2_O_2_/diesel blends because of the higher molar mass contents of hydrogen in the emulsifier. Thus, it can also be combusted practically as SD-free under a specific combustion environment^65^. Ashok and Saravan^51^ also reported similar observations for the ${\mathrm{H}}_{2}{\mathrm{O}}_{2}$ added emulsified fuel because of the presence of excess oxygen. Nevertheless, based on the supplementary molar mass of C_14_H_24_O_9_/C_3_H_6_O as an emulsifier accumulation with H_2_O_2._ Hypothetically, the least emulsified fuel probably has a significant role in reducing SD.

### Comparison of emission characteristics of H_2_O_2_/diesel blend fuel at maximum speed of engine (with and without the load)

**Figure 6:**
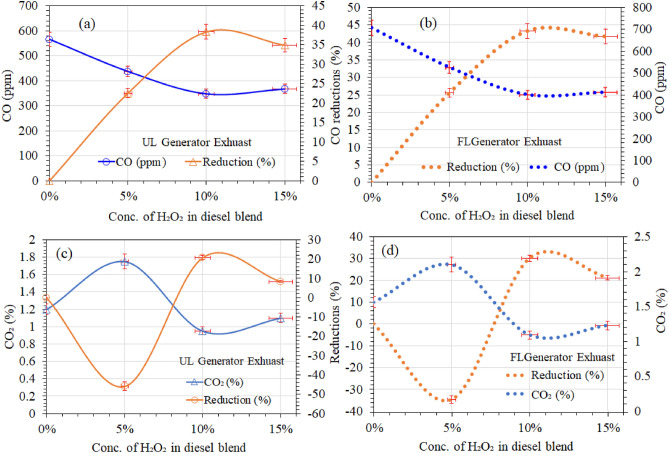
Comparison of carbon-based emissions at unloaded (UL) and full-loaded (FL) diesel engine.

**Figure 7:**
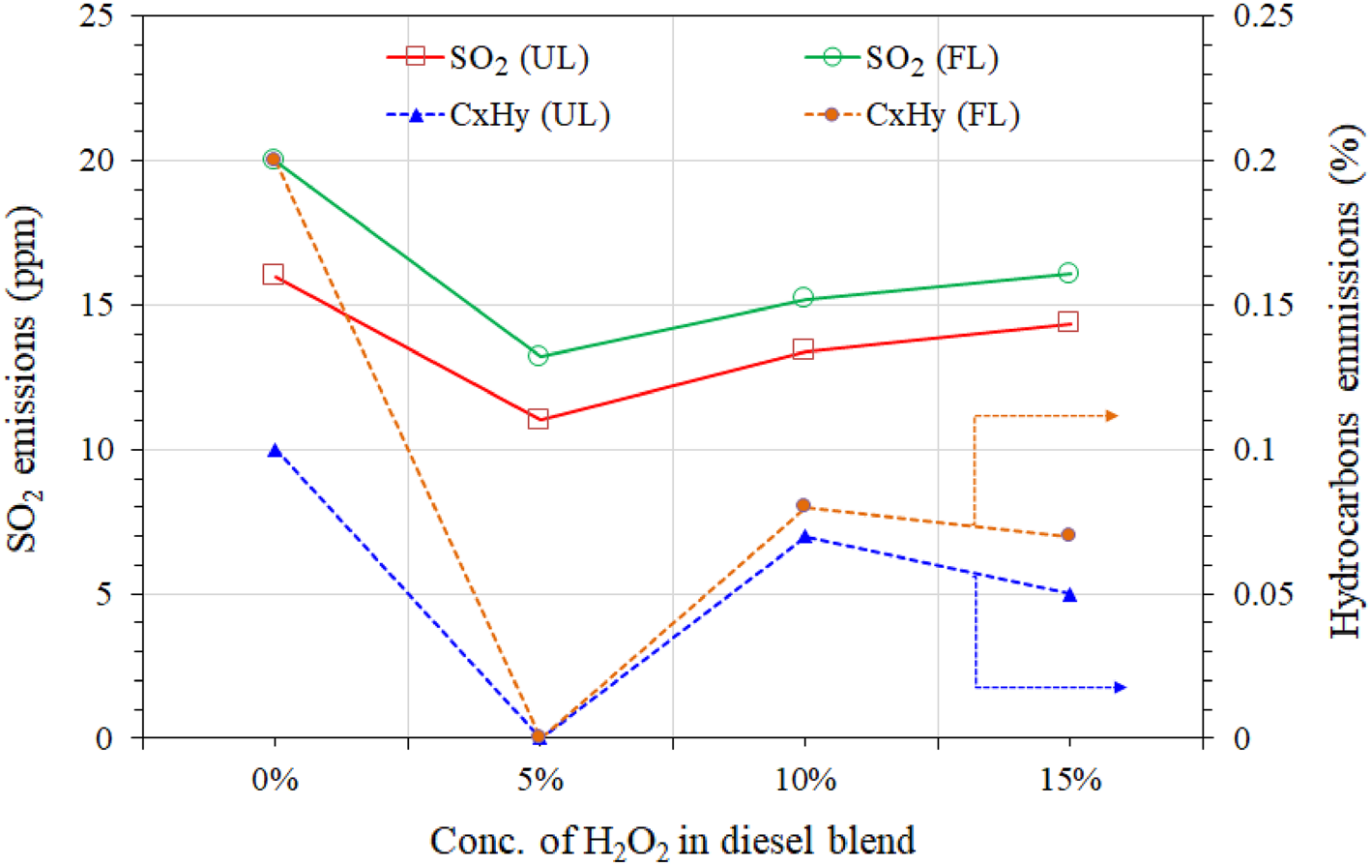
Comparison of sulfur dioxide (SO_2_) and unburned hydrocarbon emissions at unloaded (UL) and full-loaded (FL) diesel engine.

**Figure 8:**
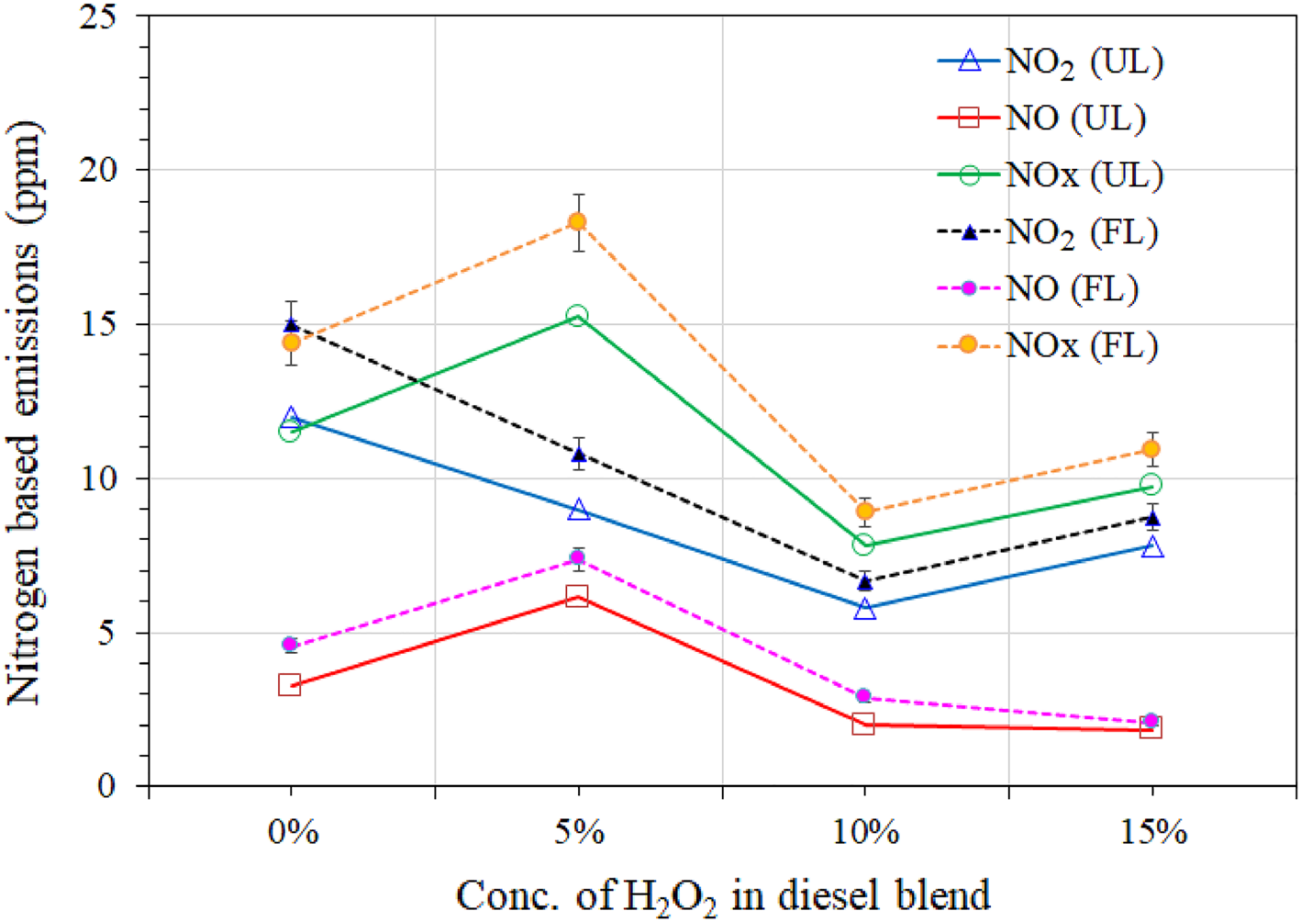
Comparison of nitrogen-based emissions at unloaded (UL) and loaded (FL) diesel engine.

**Figure 9:**
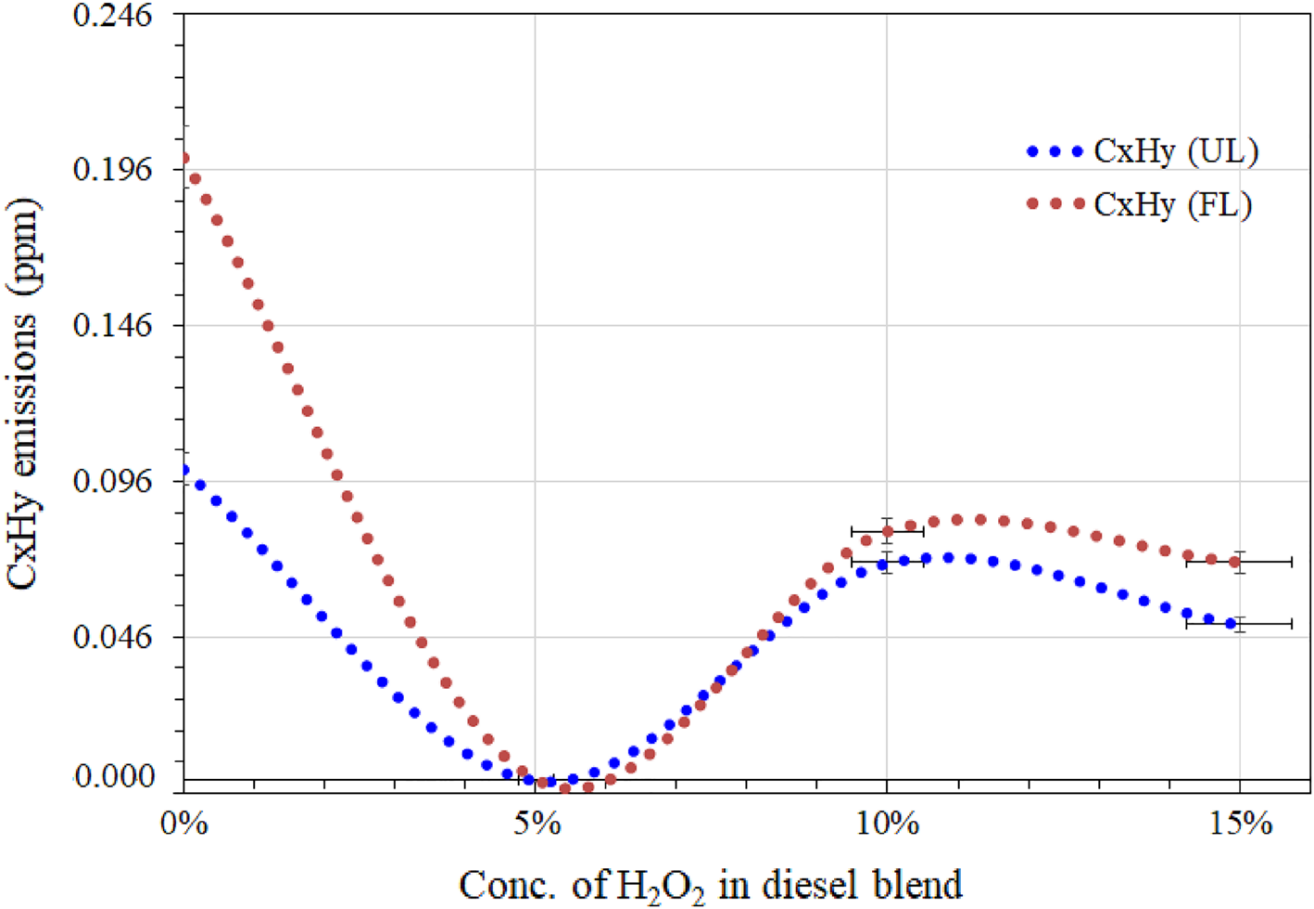
Comparison of unburned hydrocarbon (CxHy) based emissions at unloaded (UL) and loaded (FL) diesel engine.

This study evaluated exhaust emissions with the Lancom 4, a portable gas analyzer at a full-speed (2989 rpm) diesel engine with and without the load. This analyzer meets the requirements of the US EPA CTM 034 reference method with a maximum deviation of the detection limit within 2 ppm for exhaust gases and unburned hydrocarbons from 0.1 ppm^66^. Figures 6, 7, 8 and 9 summarizes that the average exhaust emission from the exhaust stream of diesel generators has been thoroughly studied. The results summarize the reduced average exhaust emissions concentrations of the loaded and unloaded generator at maximum power (see Fig. 3) after inserting 5–15 wt.% of H_2_O_2_ in the blended fuel.

Figure 6a and b summarize the emission results of CO and CO_2_ from loaded and unloaded generator exhaust, respectively. The test results (see Fig. 6a and b) of the unloaded engine revealed reference diesel (RD) CO emissions of 565 ppm and 706.25 ppm loaded, compared to 437.5 ppm and 525.4 ppm (loaded). It is generally known that diesel fuel requires more oxygen to be burned, so in the case of a fully loaded diesel engine, combustion requires a greater amount of air intake to be drowned out by each intake stroke, regardless of the position of the throttle. The air is then compressed and heated before diesel fuel is fed into the cylinder. When fuel is exposed to a higher amount of hot air, it rapidly burns. This results in a higher concentration of COx and NOx exhaust gases in the loaded engine compared to the unloaded engine. The three best diesel/H_2_O_2_ blend emulsions were evaluated in order to reduce CO content.

The UL generator shows that the 5 wt.% of H_2_O_2_ in the diesel blend represents a 22.5–25% reduction. The 10 wt.% of the diesel blend reduced CO emissions to 348.5 ppm, a 38% reduction, and the 34.8% reduction of CO emissions represents the 15 wt.% of H_2_O_2_ in the diesel blend. The 10 wt.% of H_2_O_2_ in the diesel blend shows the greatest reduction in CO emissions of the UL generator. Theoretically, the air/fuel equality ratio is defined as the difference between the definite air/fuel ratio and the stoichiometric air/diesel ratio in the compression chamber of a diesel engine^58^. In contrast, in the case of H_2_O_2_/diesel, the unstable peroxide likely provides some of the oxygen needed for the diesel to be ignited early, reducing the need for additional air in the compression chamber. Technically speaking, if the required amount of oxygen is present, then the UL diesel engines run on the leaner side of stoichiometry, CO emissions are very low in the case of an additional molar volume of peroxide in the compression chamber.

However, the loaded generator has CO emissions of about 400.7 ppm, which is 13% higher than the unloaded generator but smaller than the loaded and unloaded generators of RD fuel. It is suggested that in the case of a fully loaded diesel engine, the diesel requires more oxygen, and the probably unstable oxygen present in the diesel emulsion is probably not enough for the ignition. Therefore, the compression chamber takes in more air, and thus the contents of the CO emissions are higher than in the UL-loaded diesel engine. Nevertheless, the 15 wt.% of the diesel blends also show lower CO contents in the loaded and unloaded generator than the reference diesel but are a little higher than the 5 and 10 wt.% of the diesel blend composition, respectively.

Nevertheless, the loaded generators show almost 10–20% higher emissions than the unloaded generator in all the fuel tests. The loaded generator required more power and more fuel and air intake to be combusted, thus consequently higher the rate of CO emission. It is probably because the higher molar mass of oxygen in the diesel blend composition and higher contents of CO in the reference diesel emissions are in good agreement due to the air intake inside in-cylinder combustion. Moreover, the tendency H_2_O_2_ is entirely reactive, flaring once it has ideal environments like ignition in a closed chamber. Thus, it reacts independently and does not need any oxidizer, helping the diesel for an early and clean combustion process. But the higher amount of H_2_O_2_ in the blend yet contributes to reducing the contents of CO. Gribi et al.^67^ also found that H_2_O_2_ has individual combustion characteristics. They have reported that H_2_O_2_ can be used as a fuel or an oxidizer when reacting with other fuels, particularly in combustion chambers. Thus, it assumes the dual nature of H_2_O_2_ and explores its potential benefits in clean combustion technology.

Figure 6c and d also shows the H_2_O_2_ impact on reducing CO_2_ parts of the unloaded and loaded diesel generator’s exhaust stream. Although reference diesel had a very low CO_2_ emission (1.2%), the 5% ${\mathrm{H}}_{2}{\mathrm{O}}_{2}$ fuel blend slightly increased the CO_2_ emission to 1.75%. Similar results are also observed in loaded generators, and the CO_2_ emission exhibits higher content but is lower than the RD diesel emission, either loaded or unloaded generator. However, the values of CO_2_ emissions for higher percentages are quite like RD (1.2%), and the effect is not significantly evident on CO_2_ emission. Al-lwayzy et al.^69^ and Scragg et al.^70^ observed a small decrease in the proportion of CO_2_ in the exhaust produced by emulsion fuel containing microalgae in comparison to fuel made from biodiesel. According to Koc and Abdullah^71^, higher oxygen atom levels in the fuel mixture as a result of higher water concentrations may explain why emulsified diesel fuel has higher CO_2_ levels. The Koc and Abdullah^71^ justification could be a good agreement in the case of 5% of H_2_O_2_ in the diesel fuel blends, but the increment gap is not large with the RD diesel emission of CO_2_. Nevertheless, more experimentation is needed to explain the impact of a 5% H_2_O_2_/diesel blend on CO_2_ emissions.

Furthermore, the 10 wt.% of H_2_O_2_ in the fuel blend revealed CO_2_ contents emission totaled 0.95 percent in unloaded and 1.09 in loaded generator, representing an overall reduction of 19–21 percent from RD. Ashok and Saravanan^51^ observed similar results with diesel/ H_2_O_2_ and David and Reader^56^ (CH_4_/ H_2_O_2_) in their studies with H_2_O_2_ blended fuels, which showed a reduction of about 16.5%. However, the 15 wt.% H_2_O_2_ diesel blend slightly reduced the exhaust component of carbon dioxide in the loaded and unloaded generator.

### Comparisons of particulate matters emissions

The results of a portable gas analyzer at a full-speed (2989 rpm) diesel engine unloaded and load generator showed the influence of ${\mathrm{H}}_{2}{\mathrm{O}}_{2}$ on the fuel blends’ emission of sulfur dioxide (SO_2_). Figure 7 shows the comparison of SO_2_ emissions. The RD fuel shows higher SO_2_ emissions of 16 ppm and 20 ppm of the unloaded and loaded generator than all H_2_O_2_/diesel blends.

The H_2_O_2_/diesel blend also positively impacted the concentrations of SO_2_ exhaust stream as measured in an unloaded and loaded diesel generator. The reduced exhaust concentrations of SO_2_ are due to the substantial oxidizing property of H_2_O_2_ in the blended fuel. The 5 wt.% of H_2_O_2_ in the diesel blend shows a significant reduction of SO_2_ to 11 ppm in unloaded and 13.2 ppm in loaded generator exhaust, nearly 31.5% and 34% lower than RD diesel. Similarly, SO_2_ emissions from the 10 wt.% of H_2_O_2_ were at 13.4 ppm, and 14.35 ppm revealed the 15 wt.% H_2_O_2_ in the blended fuel. The 15 wt.% of H_2_O_2_ in the diesel blend slightly reduced *SO*_*2*_ (14.35 ppm) emissions.

Nevertheless, the unloaded and loaded generator shows considerably lower emissions of SO_2_ than RD diesel fuel. These reductions were observed due to the overall lower sulfur content of diesel fuel and no sulfur contents in H_2_O_2._ Ashok & Saravanan^51^ and David & Reader^56^ observed similar findings in their studies with H_2_O_2_ blended fuels.

The Lancom 4 portable gas analyzer has also computerized the results of nitrous oxide (NOx), nitric oxide (NO), and nitrogen dioxide (NO_2_) of exhaust emissions of reference diesel and H_2_O_2_/diesel fuel blends, and Fig. 8 summarizes the results of the comparison.

The primary mechanism causing the reduction in exhaust emissions looks like the decrease in the temperature of the combustion products as a result of vaporization of the liquid water and subsequent dilution of the gas-phase species. NOx results found positive impacts on concentrations of NO_2_ and nitrous oxide (NO) in the diesel fuel exhaust streams, either unloaded or loaded generators. Figure 8 illustrates an overall reduction comparison in nitrogen dioxide and nitrous oxide emissions due to the solid oxidizing capacity of H_2_O_2_ as it decomposes in the combustion chamber to oxygen and water.

Water produced during this reaction absorbed heat which, in turn, slightly decreased the temperature in the combustion chamber. This reduction in a temperature limited the production of NO_2_ and NO. Although reference diesel has very low emissions of NOx (12 ppm), the 5 and 10 wt.% of H_2_O_2_ in the diesel blend decreased its formation to 9 and 5.8 ppm, respectively. The reduction of NO_x_ formation caused by the combination of higher cetane number and water content reduces the diesel engine’s temperature^13,43,51^. Similar results are also observed in loaded generator emissions. The significant reduction of nitrogen-based emissions of blend fuel on unloaded or loaded generators might be a possibility of rapid vaporization and disassociation of H_2_O_2_ into hydroxyl radicles. In addition, it can also be interpreted that the H_2_O_2_ has become strenuously unstable and highly active in the combustion chamber, consequently oxidizing the NO and NO_2_ in the exhaust. Kasper et al.^68^ also investigated the significance of H_2_O_2_ on the decomposition and reduction of nitrogen-based emissions; they have experimentally demonstrated that NOx can be oxidized to NO and NO_2_ in the gas phase by OH radicals generated by the thermal decomposition of H_2_O_2_. Similar results were also observed by Saravanan et al.^72^. and Ashok & Saravanan^51^, in their studies of H_2_O_2_-diesel blends, found an overall reduction of about 18.5%.

**Figure 10:**
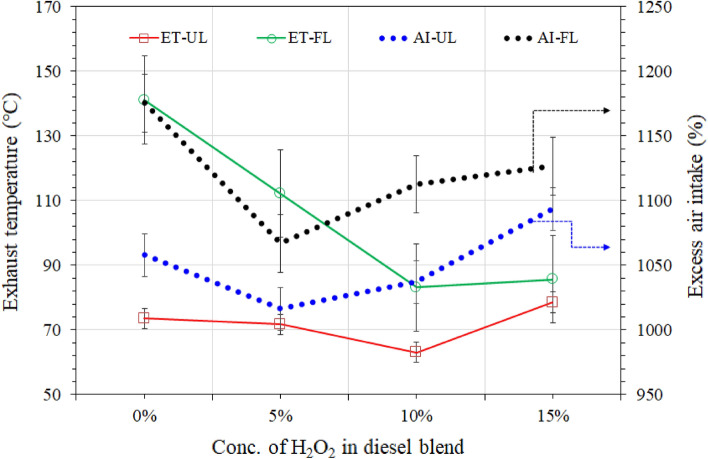
Comparison of exhaust temperature and excess air intake at unloaded (UL) and loaded (FL) diesel engine.

Figure 8 also shows the results of Nox, and it was noticed the, the 5 wt.% of H_2_O_2_ in the diesel blend shows higher NOx contents in UL and FL generater emission. It has been found that a 5% H_2_O_2_ diesel blend doesn’t make a big difference in reducing NOx and CO_2_. This is likely because there isn’t as much H_2_O_2_ in the diesel, but it does produce less heat (see Fig. 10) than RD diesel, regardless of whether the UL or FL generator. Although the temperature reduction is 2–20% in the case of 5% H_2_O_2_/diesel blend fuel exhaust, this gap is probably not enough to overcome the reduction of NOx and CO_2_. On the other hand, higher the concentrations (10–15%) of H_2_O_2_ in the diesel blend shows significant reduction in the NOx in UL and FL generator emissions. It seems that a higher water content level in the diesel blend reduces the temperature of the combustion chamber, resulting in a lower NOx concentration.

Typically, the combustion temperature, oxygen concentration, and the retention time of the combustion product in the combustion zone are often the most prevalent variables determining the amount of NOx generated. The high temperature inside the cylinder caused by the high compression ratio encourages NOx emission, and the RD results show good agreement with the experimental results^74^. The local adiabatic flame temperature is reduced by the heat of vaporization and sensible heating of water, which also reduces NOx generation. Therefore, the higher the concentration of H_2_O_2_ in the diesel blend, the greater the reduction in NOx^75^. Scrage^70^ and Koc^71^ reported similar results, which increased the water and oxygen contents while decreasing NOx and CO_2_, but the CO_2_ reduction is not yet significant. Perhaps it might be overcome in the case of the alteration of the engine.

Hydrocarbon emissions from diesel engine exhaust are also essential pollutants. The ${\mathrm{H}}_{2}{\mathrm{O}}_{2}$/diesel blends also demonstrated constructive impacts on the total hydrocarbon content of the diesel generator's exhaust stream. Figure 9 shows comparisons of the overall reduction in the concentration of unburned hydrocarbons (CxHy) due to the considerable oxidizing property of H_2_O_2_.

The RD fuel shows higher CxHy content emissions in unloaded and loaded generator exhaust. The diesel blend fuel has a 5wt.% of H_2_O_2_/diesel blend and didn’t reveal CxHy content in the unloaded and loaded generator exhaust. However, the 10 wt.% of H_2_O_2_ in the diesel blend slightly increased the production of unburned hydrocarbons. The 15% H_2_O_2_ in the blend is lower than RD diesel and the 10 wt.% H_2_O_2_/diesel blend. The lower level of unburned hydrcarbons is most likely due to the formation of acetone peroxide prior to the solution being mixed with the diesel. which most likely acts as a strong oxidizing reagent in the combustion chamber, and once diesel is ignited, this acts as a cleaning tool along with water vapors to overcome the unburned hydrocarbon reduction in the higher concentration of H_2_O_2_ in the diesel blend.

Moreover, the results revealed that, as the concentration of H_2_O_2_ in the blends increased, unstable oxygen contents improved due to the peroxiding nature of H_2_O_2_, although viscosity, density, and high heat value decreased slightly^47,73^. In general, higher density and lower viscosity lead to higher flow; thus, these findings suggested that the lower viscosity of diesel/H_2_O_2_ blend fuel could succeed in lowering fuel injection with an early ignition time^47,73^, which could result in a good agreement in the reduction of unburned hydrocarbons and NO_x_. Furthermore, the higher molar ratio of the peroxide group resulted in a drop in the viscosity of each stable blend compared to RD and a lower concentration of H_2_O_2_ in fuel blends. It also suggested that the 70% water content of H_2_O_2_ formed water droplets inside the diesel, and these droplets mixed well due to the polysaccharide polymer in the H_2_O_2_/diesel blend.

Nevertheless, unburned hydrocarbon emissions were well below those from pure diesel fuel. In terms of particulate matter (PM) emissions, the presence of water during the intensive formation of soot particles appears to significantly reduce and enhance burnout by increasing the concentration of oxidation species such as OH^73^.

Figure 10 compares the exhaust temperature of the unloaded and loaded generator at maximum power output. The exhaust temperature of RD fuel shows a higher temperature than all H_2_O_2_/diesel blend fuels either the generator is unloaded r or loaded at maximum power output. The higher exhaust temperature of RD fuel was revealed due to the higher heat of evaporation and delayed combustion process of lean diesel. However, all H_2_O_2_/diesel blend shows almost 20–41% lower exhaust temperature of the loaded generator.

Due to the higher cetane number H_2_O_2_, it has a lower latent heat of evaporation than diesel. The ignition delay for H_2_O_2_/diesel fuel diminishes, resulting in a low exhaust temperature^13,44,72^. In addition, during typical engine running, the coolant absorbs the majority of the heat. The H_2_O_2_ also has water particles, which interact with the coolant and absorb more heat, decreasing or controlling the exhaust emission temperatures^51^. The peak engine temperature constantly boosts NOx generation. Including H_2_O_2_ in the diesel blend raises the cetane rating, which precedes a reduction in ignition latency. This decreased ignition delay reduces the amount of fuel accumulated before to combustion and lowers the initial combustion rates, lowering the peak temperature and thus lowering NOx generation. Reducing NOx, COx, and CxHy in exhaust emission is a significant agreement to justify the temperature reduction^60,73^. Figure 10 also compares air intake amounts during the combustion process. Compared to RD diesel, the H_2_O_2_/diesel blend fuel shows lesser air intake in the combustion process, probably due to the availability of required oxygen in the combustion chamber.

## Methodology

### Materials

PETRONAS Sdn Bhd provided reference diesel fuel. Commercial grade H_2_O_2_ (30%) was obtained from the Chemical Company of Malaysia Berhad (CCM). The emulsifier intermediates, such as acetone (C_3_H_6_O) of 99% purity and polysaccharide polymer (agarose: C_14_H_24_O_9_), were purchased from Merck.

### Preparation of emulsifier and diesel/ H_2_O_2_ fuel blend

The emulsifier was prepared before mixing reference diesel (RD) and ${\mathrm{H}}_{2}{\mathrm{O}}_{2}$ diesel fuel blend. A polysaccharide polymer (PSP) and acetone reaction at a ratio of 1:4 w/v were accomplished in a 500-mL sealed Schott bottle. A heated magnetic stirrer mixed the solution at 50 C for 12 h. The diesel/H_2_O_2_ fuel blends were prepared with a customized solvent condensation apparatus described elsewhere^13,44,47^. During the preparation of diesel/H_2_O_2_ fuel blends, the amount of PSP emulsifier was kept at 5 vol %, and the volume ratios of H_2_O_2_ to RD varied in the range of 5–15 wt.%. Mixing the PSP emulsifier and H_2_O_2_ took 30 min to form a stable homogenized solution. As a final point, 91% of RD was inserted into the mixing vessel and kept during the mixing process until 70 min. A well-stabilized emulsion is formed utilizing the hydrophobic, hydrophilic, and lipophilic nature of the PSP emulsifier and by the sharing effects produced by the high-speed fluid stirrer in the vessel with emulsified fuel blend. All the fuel blend formulations were carried out at a constant speed of 100 rpm under variable loading *conditions* and kept the temperature of the fuel blend preparation at the ambient temperature of 25 ± 1 °C.

### Experimental setup and procedure of engine test trial for diesel blend fuel

**Figure 11:**
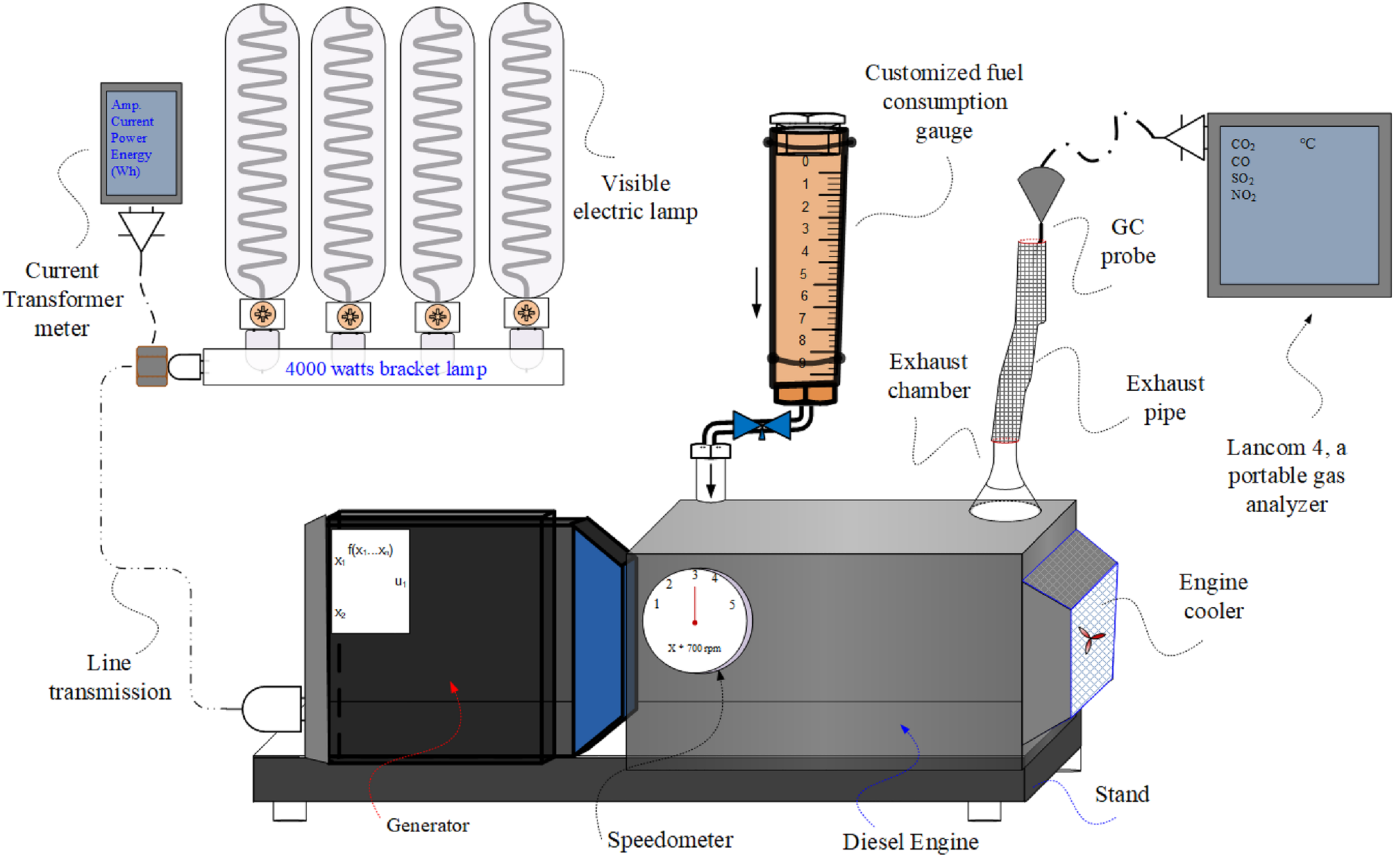
Experimental setup with Yanmar direct-inject diesel generator.

A Yanmar L48 N single-cylinder, four-stroke, direct-injection diesel engine with an output of 3.6 kW (4.7 ps) and a variable speedometer controller^53^, typically used for agricultural and residential electricity production, was the subject of the current investigation. The detailed specifications of the diesel generator are compiled in Table 1. The single-cylinder engine was chosen because it was compact and simple to maintain. The system is more suited for hot and arid circumstances because it is air-cooled, so there is no need for a radiator, water body, or pump. The test engine (a diesel generator) is shown in Fig. 11 and has been modified with four Philips 32150-5 1000 W high-intensity discharge lamps to investigate the engine load test. The load on the generator was measured using a Digital Generator Current Voltage Power Energy Meter (QV05 MK 11-380; S/N 36220526). Every measurement is taken and manually recorded. Run the engine for roughly 10 min on reference diesel fuel before starting it. The fuel flow rate was calculated using a calibrated burette and a digital stopwatch. Figure 11 displays the schematic diagram of the experimental setup together with all of the instrumentation. Before each experiment, the emission analyzer was zeroed out and calibrated for a conventional diesel engine.

**Table 1:** Specification of Yanmar L48N test engine (diesel generator)^62^.

Engine Model	L-48 N		
Type	4 stroke, vertical cylinder, air cooled diesel engine		
No. of cylinders	1		
Bore’ stroke	70′57 Mm		
Capacity	0.219 Liter		

	Maximum		Minimum
Continuous engine speed (rpm)	3600		3000
Rated output (kW)	3.6		2.8
Maximum engine speed (rpm)	3600		3000
Rated output/engine speed (kW)	3.5		3.1
High idling (rpm)	3800 ± 30		3175 ± 30
Engine weight (Dry) (Kg)	27		
Cooling system: forced air by flywheel fan			
Lubricating system: forced lubrication with trochoid pump			
Starting system: recoil start			
Overall length (L)		mm	332
Overall width (W)		mm	384
Overall height (H)		mm	417
Lubricating system	Dipstick upper limit	Litre	0.8
	Dipstick lower limit	Liter	0.55
Fuel tank capacity		Liter	2.4

### Exhaust emission analysis on diesel generator

As discussed earlier, exhaust emissions are one of the most significant problems associated with diesel fuel and thoughtfully contribute to environmental pollution. The major components of nearly all gas combustion products are N_2_, CO_2_, CO, and water vapour. They are not poisonous or toxic, although carbon dioxide has generally been recognized as a critical greenhouse gas contributing to global warming. A comparatively minute part of gas combustion products comprises undesirable toxic or lethal substances such as CO, which is formed due to incomplete combustion), hydrocarbons (appropriately indicated as ${\mathrm{C}}_{\mathrm{x}}{\mathrm{H}}_{\mathrm{y}}$ from unburned fuel, and NOx formed during elevated combustion temperatures. An AVL smoke meter is used to estimate the smoke capacity. The measurement of diesel exhaust emissions in this study was conducted with the Lancom 4—Portable Combustion and Stack Emissions Gas Analyzer. Lancom 4 analyzer satisfies the standards of the US EPA CTM 034 reference method, with a maximum detection limit variation of 2 ppm for exhaust gases and unburned hydrocarbons from 0.1 ppm. The probe of the analyzer was inserted with the exhaust stream outlet of the diesel generator to determine the amounts of the pollutants such as carbon-based emissions (CO from high range to compensated and ${\mathrm{CO}}_{2}$), nitrogen-based emissions (NO, ${\mathrm{NO}}_{2}$, NOx—calculated where ${\mathrm{NO}}_{2}$ the sensor was not fitted), ${\mathrm{SO}}_{2}$, ${\mathrm{H}}_{2}\mathrm{S}$, Hydrocarbons (${\mathrm{C}}_{\mathrm{x}}{\mathrm{H}}_{\mathrm{y}}$), respectively.

## Conclusion

In this experimental study, specific effects from the addition of hydrogen peroxide (H_2_O_2_) to diesel fuel were systematically observed for various compositions of fuel blends to discover an optimal blend that best enhances the performance of diesel fuel exhaust emissions. Due to the environmentally friendly nature of H_2_O_2_, improved ambient effects on unloaded and loaded diesel generator emissions were robustly determined and demonstrated by this study. Reduced emissions of CO, SO_2_, and unburned hydrocarbons along with NOx were achieved as the H_2_O_2_ content of the fuel blend was slightly increased. The study also demonstrated that while the addition of 5 wt.% H_2_O_2_ slightly increased the concentration of CO_2_, the amount of CO was reduced to about 25.6% for full load conditions. The number of unburned hydrocarbons (CxHy) from enhanced combustion decreased due to increased oxygen content during the combustion process. Overall, the superior environmental properties of the H_2_O_2_/diesel fuel blend were perhaps observed due to the higher cetane number potential of H_2_O_2,_ water content and adequate oxygen, which provide complete combustion with a slightly reduced temperature profile. Resulting in form of complete combustion with reduced acidic gas formations (Cox, SOx and NOx). Thus, this experimental study demonstrated that 5 and 10 wt.% of H_2_O_2_ in diesel blend fuels could be best suggested after physicochemical, thermal, and exhaust emission characterization. Therefore, this study will make an effort to contribute to the ongoing research for greener diesel fuel and to curtail the harmful greenhouse impact of conventional diesel fuel, which can contribute to reducing carbon and greenhouse emission goals.

## Supplementary Information

Supplementary Information 1.

Supplementary Information 2.

Supplementary Information 3.

Supplementary Information 4.

## Data Availability

The datasets generated during and/or analyzed during the current study are available from the corresponding author on reasonable request.
