# Supplementary material for: Influence of hydrogenated diesel/H2O2 blend fuel on diesel engine performance and exhaust emission characterization
Source: Sci Rep. 2023 Jan 16;13:836. doi: 10.1038/s41598-023-27569-5 (PMC9842606; doi:10.1038/s41598-023-27569-5)
Supplement: Supplementary file 1 — Supplementary Information 1. [file 41598_2023_27569_MOESM1_ESM.docx]

**Appendix 1:** Comprehensive core emissions from diesel engines

| **Emission**  **Factors** | **LCV** | **HDV** | **PC** |
| --- | --- | --- | --- |
| **(g/kg fuel)** | | | |
| CO | 11.71 | 10.57 | 8.1 |
| Non-Methane Volatile Organic Compounds (NMVOC) | 1.96 | 3.57 | 1.88 |
| NOx | 18.43 | 38.29 | 13.88 |
| Particulate Matter (PM) | 2.99 | 1.57 | 2.64 |
| N_2_O | 0.072 | 0.089 | 0.107 |
| NH_3_ | 0.056 | 0.018 | 0.082 |
| CO_2_ | 3.169 | 3.169 | 3.169 |
| Lead (Pb) | 1.94E^-04^ | 1.94E^-04^ | 1.94E^-04^ |
| Benzo[b]fluoranthene (BbF) | 3.69E^-05^ | 3.33E^-05^ | 5.26E^-05^ |
| Benzo[a]pyrene (BaP) | 3.19E^-05^ | 5.50E^-06^ | 4.55E^-05^ |
| Indeno[1,2,3-cd]pyrene | 2.84E^-05^ | 8.60E^-06^ | 4.05E^-05^ |
| Benzo[k]fluoranthene (BkF) | 3.21E^-05^ | 3.72E^-05^ | 4.58E^-05^ |

LCV: light commercial vehicles; HDV: heavy-duty vehicles; PC: passenger cars
